# Phosphatidylserine externalized on the colonic capillaries as a novel pharmacological target for IBD therapy

**DOI:** 10.1038/s41392-021-00626-z

**Published:** 2021-06-16

**Authors:** Xuerui Zhang, Lulu Song, Lin Li, Banghui Zhu, Lina Huo, Zhaoqing Hu, Xinran Wang, Jie Wang, Mengyue Gao, Jing Zhang, Zichun Hua

**Affiliations:** 1 The State Key Laboratory of Pharmaceutical Biotechnology, School of Life Sciences, Nanjing University, Nanjing, China; 2 Changzhou High-Tech Research Institute of Nanjing University and Jiangsu Target Pharma Laboratories Inc, Changzhou, China; 3 School of Biopharmacy, China Pharmaceutical University, Nanjing, China

**Keywords:** Molecular medicine, Biochemistry

## Abstract

Inflammatory bowel disease (IBD) is a chronic and relapsing disorder for many people associated with poor health. Although there are some clinical drugs for IBD treatment, the development of effective therapeutics on IBD patients has always been necessary. Here, we show that externalized phosphatidylserine (PS) is observed on the surface of colonic capillaries. Annexin A5 (ANXA5) with high affinity for PS has a good targeting to the colon and effectively alleviates experimental colitis. In contrast, ANXA5 mutant (A5m) lacking the PS-binding ability, has no accumulation in the colon and no therapeutic effects on colitis. Mechanistic investigations indicate that ANXA5 reduces the inflammatory cell infiltration by inhibiting endothelial cell activation dependent on PS-binding ability. With the increasing of PS exposure on activated HUVECs (human umbilical vein endothelial cells), ANXA5 binding induces the internalization of TLR4 via PS-dependent endocytosis. We provide new insights on the molecular mechanism of ANXA5 for its anti-inflammatory effect. Our data suggest that PS-externalization is a potential target of ANXA5 aiming at targeted drug delivery (TDD) for IBD treatment.

## Introduction

Inflammatory bowel diseases (IBDs) are chronic relapsing disorders of the gastrointestinal tract, characterized pathologically by intestinal inflammation and epithelial injury, known as ulcerative colitis (UC), Crohn’s disease (CD), and indeterminate colitis (IC)^1^. The therapy of IBD is dominated by the administration of anti-inflammatory and immune-modulating drugs, which suppress the intestinal inflammation and thus improve disease-related symptoms. Blockade of tumor necrosis factor (TNF) is commonly used as a standard therapy for IBD in clinic, resulting in blockage of pro-inflammatory signals or molecules that are upregulated by TNF-α^2^. But long-term therapeutic use of anti-TNF has adverse effects, including potential for development of skin lesions, immune reactions, infections, and cancers^3^. Two monoclonal antibody drugs, Natalizumab and Vedolizumab, against α4 integrin are currently available for the treatment of IBD. Continuing Natalizumab therapy is associated with an increased risk of progressive multifocal leukoencephalopathy (PML)^4^. Vedolizumab has a favorable safety profile with low incidence rates of serious infections, infusion-related reactions, and malignancies^5^. But for patients with IBD, the clinical problems have not been solved and need the development of innovative drugs and new treatment strategies.

Targeted drug delivery (TDD) is a strategy to effectively treat disease with minimal detrimental side-effects^6^. In normal cells, phosphatidylserine (PS) is asymmetrically distributed across the plasma membrane, exclusively localized to the inner leaflet^7^. In stressed or dying cells, PS externalization as a cell surface fingerprint might be a biomarker indicating the lesion area for TDD^8^. For example, PS-targeting antibodies can bind to exposed PS on cell surface of tumor vascular endothelium, enabling antibody-dependent cell-mediated cytotoxicity^9^. A chimeric antibody, bavituximab targeting the exposed PS of virus-infected cells rescued mice from lethal mouse cytomegalovirus infections, indicating side-out PS as a therapeutic strategy for viral diseases^10^. Notably, Diannexin (a homodimer of ANXA5) as PS-targeting agent has entered clinical trials in patients with kidney transplants^11^.

A potential trigger for PS exposure is external stress, such as hypoxia, inflammation, and infection^10,12,13^. Many of these stressors are present in the enteritis^14,15^, so it is possible for the occurrence of externalized PS in the colon. To test this hypothesis, ANXA5, a Ca^2+^ and phospholipid-binding protein^16^, is used for verification. With the high affinity for PS, ANXA5 can bind to aged erythrocytes, activated platelets, endothelial microparticles, and tumor vascular, for the exposed PS on their surfaces. Surface-expressed PS constitutes a target for apoptosis imaging using ANXA5 in vitro^17^ or in vivo in animal models^18,19^ and patients^20,21^. In addition, ANXA5 has a potential inhibitory effect on inflammation. In vivo ANXA5 dampens inflammation when administered to various mouse models^8,22–24^. Administration of ANXA5 reduces inflammation and suppresses plaque development in a PS-dependent manner^25^.

Considering the PS targeting and anti-inflammatory effects of ANXA5, we investigate the possibility of ANXA5 as a PS-targeting agent on IBD therapy. In this study, we demonstrate that externalized PS is indeed exposed on the vascular endothelium of colonic capillaries. ANXA5 effectively alleviates TNBS-induced colitis by inhibiting inflammatory cell infiltration. Our finding suggests that PS-targeting delivery of ANXA5 to colonic capillaries has potential application values in the treatment of IBD.

## Results

### Exposure of phosphatidylserine on the capillaries of colonic mucosa

**Fig. 1:**
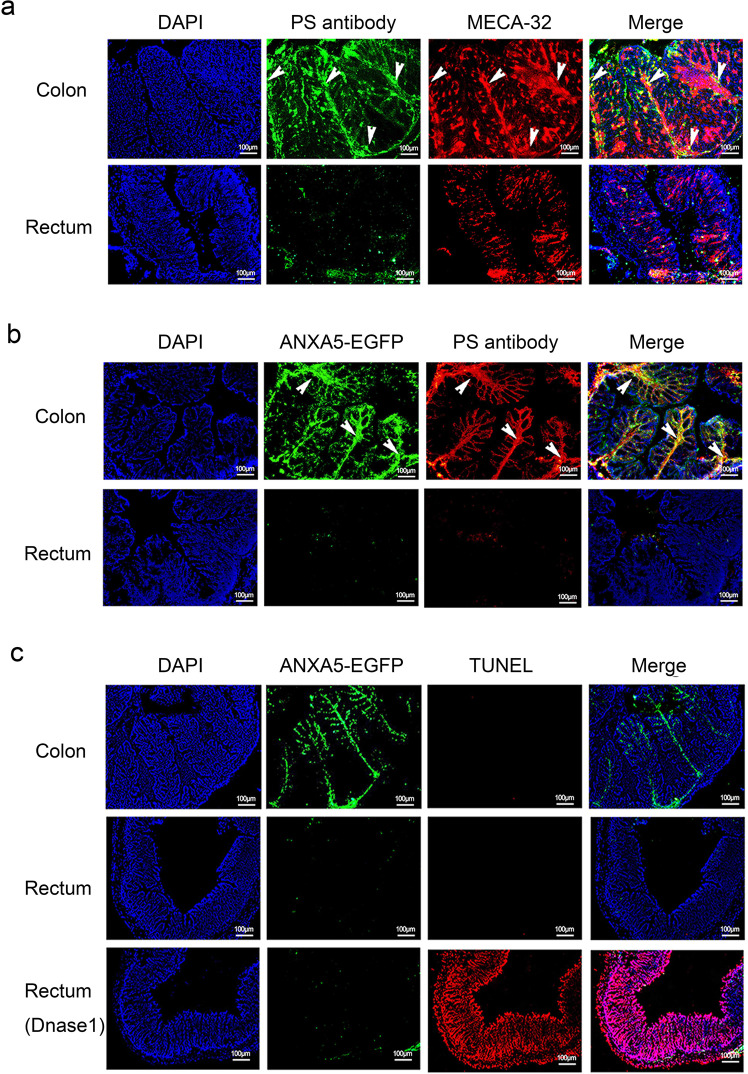
PS exposure on the surface of colonic capillaries. **a** Normal C57BL/6 mice were received anti-PS antibody by tail vein injection. The sections of colon and rectum were stained for anti-PS antibody in green and MECA-32 in red. Representative images showed the staining of exposed PS co-localized with MECA-32. **b** Mice were injected intravenously with ANXA5-EGFP and anti-PS antibody. The sections of colon and rectum were prepared after 30 min of injection. Anti-PS antibody was stained in red with AF594-conjugated secondary antibody. The yellow in merged image indicate the co-location of ANXA5-EGFP and anti-PS antibody. **c** After intravenous injection of ANXA5-EGFP, the intestinal sections were prepared for TUNEL assay. Dnase I treatment as a positive control. Data are representative of more than three experiments (*n* = 3 mice/group). Scale bars: 100 μm. Arrows pointing to the co-location of dual fluorescences

To test the possibility of PS exposed on the capillary endothelium of colon, normal C57BL/6 mice were administered with the primary antibody (anti-PS antibody versus control IgG) by intravenous injection. Thirty minutes after the injection, colonic sections were prepared for dual immunostaining of exposed PS and MECA-32, a marker specific for mouse vessel endothelia^26^. The positive staining of MECA-32 showed a network of interconnected vessels in the colonic section. The staining of exposed PS was also observed on these vessels, and co-localized with MECA-32 staining (Fig. 1a), indicating the PS exposure on the vascular endothelium of colon. Then ANXA5-EGFP as a PS imaging agent was used to further validation. After tail vein injection of ANXA5-EGFP and anti-PS antibody, immunofluorescence observation showed that ANXA5-EGFP had a good co-localization with anti-PS antibody in the colon (Fig. 1b). As negative controls, injection of normal IgG or EGFP protein had no fluorescence signal in the colonic sections (Supplementary Fig. S1). In addition, we also observed the special fluorescence signal of ANXA5-EGFP in the jejunum and ileum, less in rectum (Supplementary Fig. S2). Finally, TUNEL assays were performed for in situ detection of apoptotic signals in the colon. TUNEL signals were only in the positive control with Dnase I treatment, not in the normal colon and rectum (Fig. 1c). Therefore, ANXA5-EGFP binding to colon is not indicative of tissue damage or cell death. The externalized PS on the capillaries of colonic mucosa provides the sites for targeted delivery of ANXA5 to the colon.

### The protein properties of ANXA5 and its mutant A5m

**Fig. 2:**
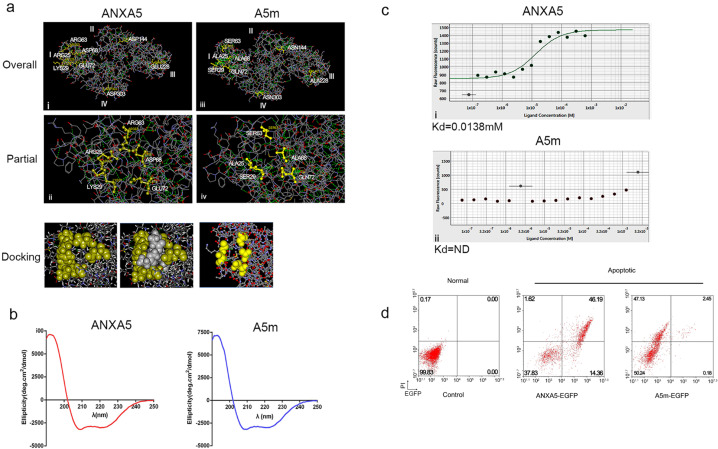
The mutant A5m is an inactive analogs of ANXA5. **a** Molecule modeling of ANXA5 and A5m protein. i, iii: overall view of protein conformation; ii, iv: partial view of PS-binding region. The key amino acids responsible for PS-binding in yellow. The PS-binding pocket of ANXA5 is surrounded by R25, K29, R63, D68, and E72 (ii), but mutated in A5m (iv). Using DS docking software (DS2.4), the docked conformation of PS matches the binding pocket of ANXA5, and mismatches with A5m. **b** The protein conformations of ANXA5 and A5m showed similar structural signals detected by Circular Dichroism (CD) spectrum. **c** MST analysis of PS liposomes binding on ANXA5-EGFP and A5m-EGFP in the presence of 2.5 mM Ca^2+^. Data are representative of three independent experiments. ND, not determined. **d** Apoptotic cells were co-stained with PI and ANXA5-EGFP or A5m-EGFP. Normal cells A549 as negative controls. Representative data are from three independent experiments

To verify the specificity of recombinant ANXA5 binding to PS, we constructed an ANXA5 mutant (A5m) as a dominant-negative control of ANXA5, which is lack PS-binding ability^27,28^. The crucial amino acids required for PS-affinity binding were mutated as R25A, K29S, R63S, D68A, E72Q, D144N, E228A, and D303N in the A5m mutant. Molecule modeling showed that five amino acids at R25, K29, R63, D68, and E72 in domain I encircle a binding pocket for PS, whereas mutations on these sites totally destroy the conformation for PS binding (Fig. 2a). Both ANXA5 and its mutant A5m were prepared with the final purity over 98% (Supplementary Fig. S3a, b). There were no significant differences in their protein stability (Supplementary Fig. S3c). The Circular Dichroism (CD) spectrum is used to analyze the secondary structural features of protein such as α-helix and β-sheet. CD signals showed the identical conformation of protein structure between two proteins (Fig. 2b). But in the measurement of PS affinity by microscale thermophoresis (MST), the affinity of PS liposomes on ANXA5 protein was detectable, not on A5m protein (Fig. 2c). The failure of A5m on PS binding was further examined by apoptosis detection. FACS analysis showed no binding of A5m-EGFP on apoptotic cells (Fig. 2d). Therefore, A5m as an inactive analog of ANXA5 is a perfect control for studies on PS-binding activity.

### ANXA5 targeting to the colon in a PS-dependent manner

**Fig. 3:**
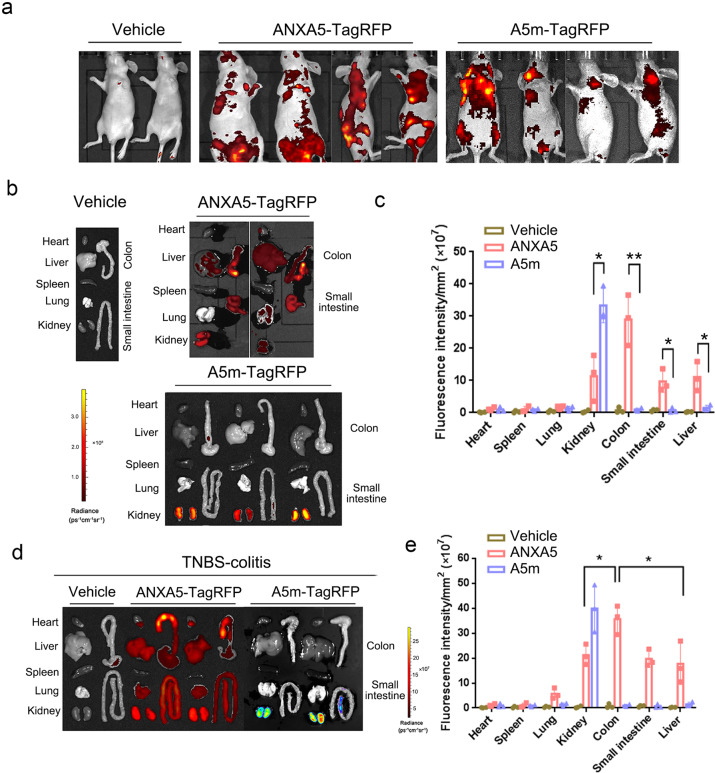
ANXA5 targeting to the colon in a PS-dependent manner. **a** Nude mice were injected with ANXA5/A5m-TagRFP by tail vein (*n* = 3). After 30 min, in vivo imaging of fluorescent proteins was visualized by IVIS Lumina XR system. **b** Ex vivo imaging of ANXA5/A5m-TagRFP in various tissues was visualized after dissection. Red to yellow color indicates increased fluorescence intensity. Fluorescence intensity of various tissues was quantitatively analyzed in (**c**). *P < 0.05, **P < 0.01 versus the control group according to Student’s *t* tests. **d** BALB/c mice with TNBS colitis were injected with ANXA5/A5m-TagRFP by tail vein (*n* = 3). After dissection, Ex vivo imaging of ANXA5/A5m-TagRFP in various tissues was visualized, and quantitatively analyzed in (**e**). Data are represented as mean ± SD. *P < 0.05 versus the control group according to Student’s *t* tests

To determine the PS-targeted delivery of ANXA5 to intestinal tissues, ANXA5-TagRFP was injected into nude mice by the tail vein for in vivo imaging. The strong fluorescence of ANXA5-TagRFP was concentrated in the abdominal cavity compared to A5m-TagRFP administration (Fig. 3a). After dissection, Ex vivo fluorescence of ANXA5-TagRFP was stronger in the colon than in liver and kidney, while A5m-TagRFP mainly in the kidney (Fig. 3b). Quantitative analysis of fluorescence intensity indicated the distinct distribution of ANXA5-TagRFP in intestinal tissues, especially in the colon (Fig. 3c). Compared to A5m-TagRFP, these results demonstrated that the colonic binding of ANXA5 is dependent on PS-binding activity.

Next, we examined the distribution of ANXA5-TagRFP in mice with colitis. TNBS-induced colitis was established in BALB/c mice. ANXA5-TagRFP or A5m-TagRFP was injected by tail vein into colitic mice, respectively. Similarly, the fluorescence intensity of ANXA5-TagRFP was stronger in the colon, and A5m-TagRFP in the kidney (Fig. 3d). However, in colitic mice, more ANXA5-TagRFP was observed in the liver and kidney (Fig. 3e), probably because of more PS exposure on vascular endothelial cells triggered by inflammatory signal. Together, the targeted delivery of ANXA5 to colon is PS-dependent.

### ANXA5 ameliorates TNBS-induced colitis in mice

**Fig. 4:**
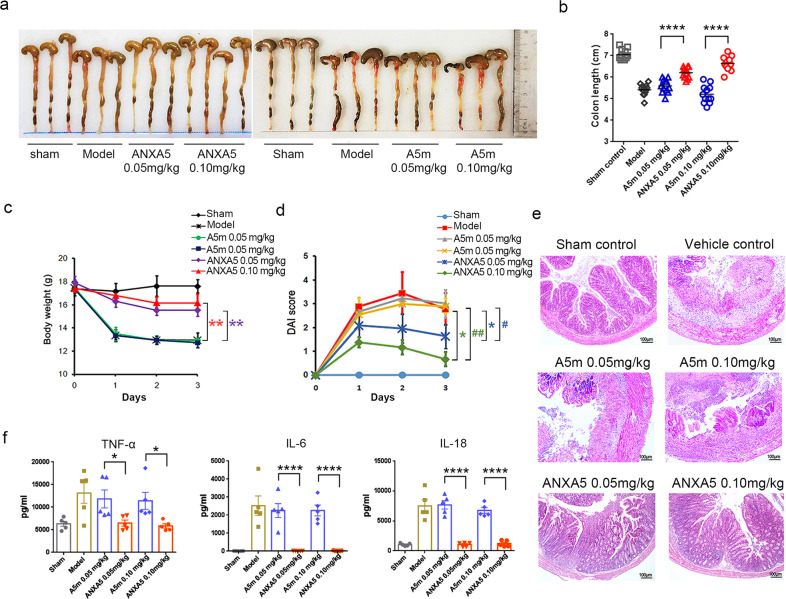
ANXA5, not A5m, alleviates TNBS-induced colitis. Mice with TNBS-induced colitis were treated with ANXA5 or A5m administration, respectively (*n* = 10 mice per group). Clinical features and severity were monitored by colon photographs (**a**), colon lengths (**b**), body weight loss (**c**), and disease activity index (DAI) (**d**). *P < 0.05; **P < 0.01 versus the model group according to Student’s *t* tests. ^#^*P* < 0.05; ^##^*P* < 0.01 versus the corresponding A5m group at the same dosage according to Student’s *t* tests. **e** Representative images of colonic sections by H&E staining. **f** The inflammatory cytokines expressions of TNF-α, IL-6, and IL-18 in the serum (*n* = 5/group). Data are presented as means ± SD. *P < 0.05; **P < 0.01; ****P < 0.0001 versus the corresponding control group according to Student’s *t* tests

To investigate the therapeutic effects of ANXA5 on colitis, mouse model of TNBS-induced colitis was used in the assessment. By morphological observation, the phenotype of enteritis was observed in model group and A5m group, but the colons of ANXA5 group showed no edema and no hyperemia (Fig. 4a). Consistently, the shortening of colon and loss of body weight were also alleviated by the treatment of ANXA5, not by A5m treatment (Fig. 4b, c). The disease activity index (DAI) indicated the protective role of ANXA5 treatment in TNBS-induced colitis (Fig. 4d). By H&E staining, colonic sections of A5m group showed severe pathological features, including loss of goblet cells, distortion of crypts, mucosal damage, and necrosis, but less pathological changes in ANXA5 group (Fig. 4e). The increase of inflammatory factors in plasma is commonly related to the severity of colitis^29,30^. Compared with A5m group, ANXA5 treatment significantly reduced the production of inflammatory cytokines, such as TNF-α, IL-6, and IL-18 (Fig. 4f). Together, these results indicated that the PS-binding ability of ANXA5 was critical for the anti-inflammatory effect in TNBS-induced colitis.

### ANXA5 reduces the infiltration of inflammatory cells in colitis

**Fig. 5:**
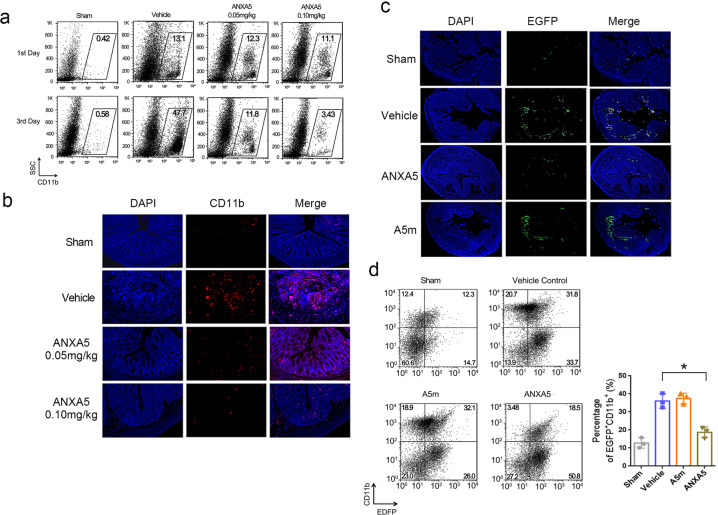
ANXA5 inhibits the inflammatory cell infiltration. **a** Mice with TNBS induction were treated with ANXA5 at two different doses. Leukocytes in colonic mucosa were collected on day 1 and day 3, respectively, and stained with CD11b-APC for FACS analysis. **b** On day 3 after TNBS induction, colonic sections were stained with CD11b-APC (red) and DPAI (blue). **c** TNBS-colitic mice were pretreated with ANXA5 or A5m, and then received EGFP^+^ inflammatory cells derived from EGFP mice. The infiltration of EGFP^+^ cells was inhibited by ANXA5, not by A5m treatment. **d** The percentages of EGFP^+^CD11b^+^ in the colon were analyzed by FACS. Data are representative of three independent experiments shown as mean ± SD (*n* = 3 mice per group). *P < 0.05 versus the vehicle control group according to Student’s *t* tests

Since inflammatory cell infiltration contributes to TNBS-induced colitis^31^, we then explored the effects of ANXA5 in this process. CD11b is a typical marker used for an indicator of neutrophils, macrophages, and monocytes^32^. On the 1st day after TNBS induction, the leukocytes of colonic mucosa were obtained and analyzed by FACS. A small amount of CD11b-positive cells were detected in each group with no significant difference (Fig. 5a). But on the 3rd day of TNBS induction, ANXA5 treatment effectively inhibited the infiltration of CD11b-positive cells, especially ANXA5 (0.10 mg kg^−1^) group versus vehicle group (3.43 ± 0.75% vs 44.85 ± 4.78%, *P* < 0.01) with significant differences (Fig. 5a). The reduced infiltration of CD11b-positive cells with ANXA5 treatment was also observed by immunofluorescence assay (Fig. 5b). For more direct validation, inflammatory cells in abdominal fluid from EGFP mice were prepared for the injection into colitic mice. Mice with TNBS colitis were pretreated with ANXA5 or A5m administration, and then injected intravenously with EGFP^+^ inflammatory cells. As expected, the infiltration of EGFP^+^ inflammatory cells in the colon was effectively inhibited by ANXA5 treatment, not by A5m treatment (Fig. 5c). FACS analysis further showed that the infiltration of EGFP^+^CD11b^+^ cells was reduced by ANXA5 treatment (vehicle group, 36.07 ± 2.313%; ANXA5 group, 18.73 ± 1.729 %, *P* < 0.05) (Fig. 5d). These results demonstrate that the anti-inflammatory effect of ANXA5 is due to its inhibition on inflammatory cell infiltration in colitis.

### ANXA5 inhibits LPS-induced endothelial activation dependent on its PS binding

**Fig. 6:**
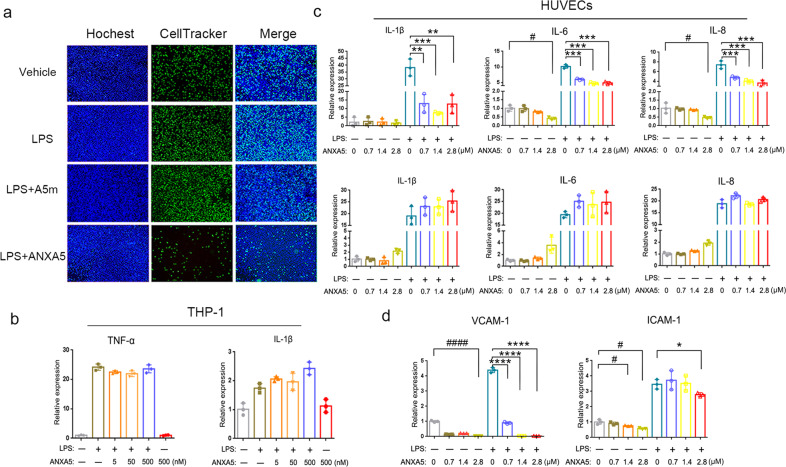
ANXA5 inhibits monocyte-endothelial cell adhesion and HUVECs activation. **a** Representative images of monocyte (THP-1) adhesion to endothelial cell (HUVEC). HUVECs were pretreated with LPS (0.2 μg/ml) or ANXA5/A5m (250 nM) as indicated, and then incubated with THP-1 cells (labeled with CellTracker in green). Hoechst staining for total cells. **b** THP-1 cells were induced by LPS (10 ng/ml) and treated with ANXA5 (0–500 nM). TNF-α and IL-1β mRNA were detected by qRT-PCR. **c** HUVECs were activated by LPS (1 μg/ml) and treated with ANXA5 or A5m at different doses. The levels of cytokines (IL-1β, IL-8, and IL-6) were analyzed by qRT-PCR. **d** The levels of ICAM-1 and VCAM-1 mRNA in LPS-activated HUVECs treated with ANXA5. All data are presented as mean ± SD representative of three independent experiments. *P < 0.05, **P < 0.01, ***P < 0.001, ****P < 0.0001, ^#^*P* < 0.05, ^####^*P* < 0.0001 versus the corresponding control group according to Student’s *t* tests

The infiltration of leukocytes from the vessel to a site of inflammation requires the interaction with endothelial cells. To test the effect of ANXA5 on leukocyte adhesion to endothelial cells, we performed in vitro assay on monocyte-endothelial cell interaction using HUVECs and THP-1 cells. LPS-activated HUVECs were pretreated with ANXA5 or A5m protein, and then co-incubated with THP-1 cells. The cell adhesion of THP-1 on HUVECs was significantly inhibited by ANXA5 treatment not by A5m treatment (Fig. 6a), consistent with the above observation on cell infiltration (Fig. 5). In LPS-activated THP-1 cells, ANXA5 treatment had no effect on the induction of inflammatory cytokines TNF-α and IL-1β (Fig. 6b). However, in LPS-activated HUVECs, the levels of IL-1β, IL-6, and IL-8 mRNA were significantly inhibited by ANXA5 treatment (Fig. 6c). Similarly, ANXA5 treatment also reduced the level of MIP-1α/CCL3, a key chemokine for monocytes/macrophages infiltration (Supplementary Fig. S4). Because A5m had no effect on these cytokines, the result suggested the inhibitory effect of ANXA5 on HUVEC activation dependent on its PS-binding ability. Vascular cell adhesion molecule-1 (VCAM-1) and intercellular cell adhesion molecule-1 (ICAM-1) as important adhesion molecules, their expressions on vascular endothelial cells are essential for leukocyte trafficking into inflammatory sites. In LPS-activated HUVECs, both VCAM-1 and ICAM-1 expressions were reduced by ANXA5 treatment (Fig. 6d), providing a possible mechanism for the inhibitory effect of ANXA5 on inflammatory cell infiltration.

### ANXA5 induces the internalization of TLR4 dependent on PS-positive membrane

**Fig. 7:**
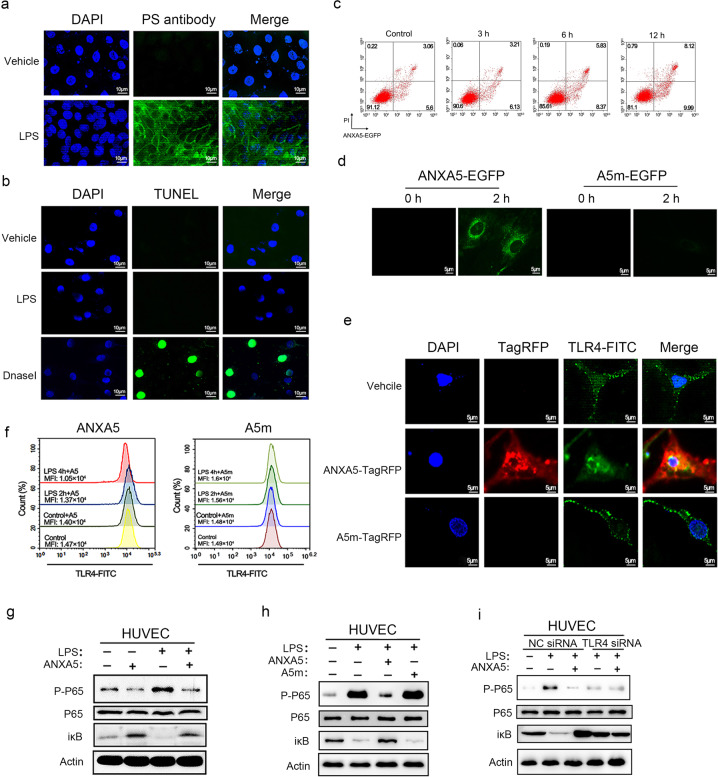
ANXA5 induces the internalization of TLR4 via a PS-dependent entry. **a** PS externalization is present in LPS-activated HUVECs. Living HUVECs activated by LPS (1 μg/ml) were incubated with anti-PS antibodies. Then HUVECs were stained by AF488-conjugated secondary antibody. No LPS treatment as a negative control. Scale bars: 10 μm. **b** LPS-activated HUVECs were examined by TUNEL assay. Dnase I treatment as a positive control. **c** HUVECs were treated with LPS for different times. The exposed PS on activated HUVECs was detected by ANXA5-EGFP (50 μg/ml) and analyzed by FACS analysis. **d** Direct fluorescence observation of living HUVECs incubated with ANXA5/A5m-EGFP. After 2 h of ANXA5/A5m-EGFP incubation, HUVECs were observed by fluorescence microscope. **e** LPS-activated HUVECs were activated by LPS and then incubated with TLR4-FITC antibody. In the absence of ANXA5-TagRFP, the fluorescence signal of TLR4-FITC was spotty mainly on the plasma membrane. When the addition of ANXA5-TagRFP, TLR4-FITC was observed inside cells and co-localized with ANXA5-TagRFP. A5m-TagRFP as a negative control. **f** HUVECs were activated with LPS (1 μg/ml) for 0, 2, 4 h, and then treated with ANXA5 (250 nM) or A5m (250 nM) for 30 min. The surface TLR4 was stained by TLR4-FITC antibody for FACS. Data are presented as mean value of MFI representative for three independent experiments. **g** The activation of NF-κB in HUVECs treated with LPS and ANXA5 as indicated. P65 phosphorylation and IκB degradation were analyzed by western blot. **h** HUVECs were treated with different stimulation as indicated and were analyzed by western blot with antibodies against p65 and iκB. **i** HUVECs were transfected with NC siRNAs or TLR4 siRNAs. After stimulation with LPS and ANXA5, NF-κB activation was analyzed by western blot

To figure out the molecular mechanism of ANXA5 function on endothelial cell activation, the exposure of PS was further examined in LPS-activated HUVECs. By incubation of anti-PS antibody with activated HUVECs, positive staining of PS exposure was analyzed by immunofluorescence analysis. The result showed no positive staining of anti-PS antibody in normal HUVECs, but strong fluorescence signal in activated HUVECs (Fig. 7a). There were no apoptotic signals in activated HUVECs by TUNEL assay (Fig. 7b), which is consistent with previous observation that PS exposure on the capillaries of colon is not associated with apoptosis. With the prolongation of LPS stimulation, there was an increasing binding of ANXA5-EGFP to HUVECs by FACS analysis (Fig. 7c). In addition, no increased binding of ANXA5-EGFP was observed inactivated THP-1 cells (Supplementary Fig. S5), consistent with the result that ANXA5 has no effect on THP-1 cell activation (Fig. 6b).

Recent studies have reported that the externalization of PS is a novel portal of cell entry^33,34^. The ANXA5-PS-mediated endocytic pathway was reported in living tumor cells and activated cells^35,36^. We speculate that the endocytosis might be triggered by ANXA5 binding to PS in activated HUVECs. To test this hypothesis, we traced the fluorescence of ANXA5-EGFP after incubation with activated HUVECs. After 2 h incubation, fluorescent spots actually occurred inside the cells, suggesting the entry of ANXA5-EGFP into activated HUVECs (Fig. 7d). As a negative control, no GFP fluorescence was observed in HUVECs with A5m-EGFP incubation. These results indicate the ANXA5-PS-mediated endocytosis present in endothelial cells.

ANXA5-PS-mediated endocytosis leads to the internalization of the embedded proteins in PS-exposure membrane, such as tissue factor^37^. Toll-like receptor 4 (TLR4) is a critical receptor for LPS and activates inflammatory responses^38^. To determine whether TLR4 internalization is involved in the ANXA5-PS-mediated endocytosis, FITC-labeled anti-TLR4 antibody (TLR4-FITC) was used to trace the translocation of TLR4. The green fluorescence of TLR4-FITC was observed mainly on the cell membrane of activated HUVECs. However, the addition of ANXA5-TagRFP induced the massive internalization of TLR4-FITC and co-localized with TLR4-FITC in the cytosol, whereas A5m-TagRFP treatment had no effect on TLR4 localization (Fig. 7e). Furthermore, the surface TLR4 stained by TLR4-FITC was also measured by FACS analysis. In ANXA5 treatment group, the fluorescence intensity of TLR4-FITC was gradually decreased with the prolongation of LPS stimulation, not in A5m treatment (Fig. 7f). As the main intracellular response to TLR4, NF-κB activation was detected by western blot. Both P65 phosphorylation and IκB-α degradation in LPS-activated HUVECs were inhibited by ANXA5, not by A5m (Fig. 7g, h). When endogenous TLR4 knockdown by TLR4 siRNA (Supplementary Fig. S6), ANXA5 treatment showed no effect on NF-κB activation in HUVECs (Fig. 7i). These results suggest that the anti-inflammatory mechanism of ANXA5 is to downregulate surface TLR4 via the PS-dependent endocytosis.

## Discussion

PS-externalization is the most prominent characteristic of apoptotic cells, which is a well-explored phenomenon to image cell death for diagnostic purposes^39–41^. PS and its binding ligands also have potential applications in treatment of a variety of diseases including cardiovascular diseases and cancer^8^. In addition to apoptotic cells, PS exposure is also associated with oxidative stresses, aging, and infections. In endothelial cells, PS exposure can be triggered by hypoxia, thrombin, inflammatory cytokines, and hydrogen peroxide. All of these triggers can lead to PS exposure in the absence of necrosis or apoptosis^42^. Many of these stressors are known to be present in enteritis. In this paper, we demonstrate the exposure of PS in the capillaries of colonic mucosal. Using a modified IHC technique (intravenous injection of a primary antibody for exposed PS binding), PS exposure was detected in the endothelium of colon, similar to externalized PS on tumor vasculature. As a high PS-affinity protein, ANXA5 administration was concentrated in the colonic distribution. PS exposure provides the basis for targeted delivery of ANXA5 in the treatment of colitis.

**Fig. 8:**
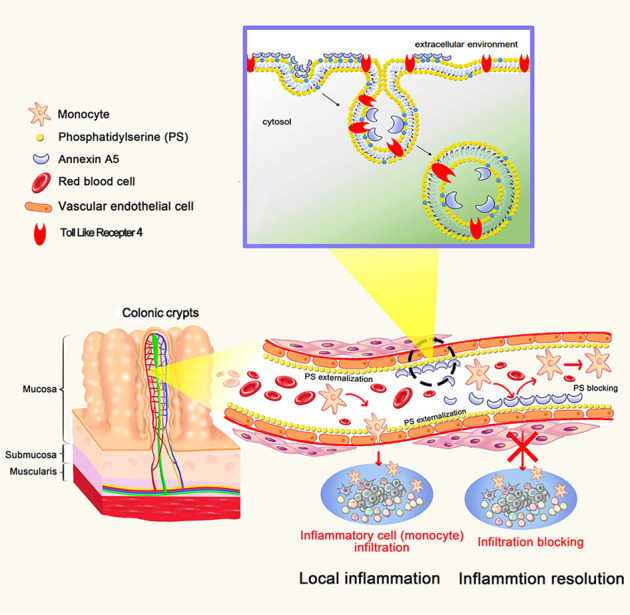
The schematic diagram of therapeutic effects of ANXA5 in colitis. Externalized PS on the surface of colonic capillary lead to the targeted delivery of ANXA5 to colon. ANXA5 alleviates colitis by reducing inflammatory cell infiltration. Mechanistic investigations indicate that ANXA5 binds the exposed PS of activated HUVEC and inhibits its activation by triggering the internalization of TLR4 via a PS-dependent pathway

The infiltration of inflammatory cells plays an important role in the pathogenesis of IBD. The interaction of blood leukocytes with endothelial cells is initially induced by adhesion molecules VCAM-1 and ICAM-1. The increased expressions of VCAM-1 and ICAM-1 were reported in the colon of patients with IBD^43–46^. Our results showed that ANXA5 treatment effectively inhibited early inflammatory cell recruitment, adhesion, and infiltration (Figs. 5 and 6). Mechanistic investigations indicated that ANXA5 induced the internalization of TLR4 in a PS-dependent endocytosis. Downregulation of surface TLR4 by ANXA5 attenuated the activation of NF-κB, subsequently inhibited LPS-induced HUVEC activation, including the induction of cytokines, chemokines, adhesion molecules VCAM-1 and ICAM-1. Our investigation proposes a novel anti-inflammation mechanism of ANXA5 in the initiation of inflammatory response (Fig. 8).

Because most of the adhesion molecules are upregulated in inflammatory bowel diseases, therapeutic compounds have been designed directly against trafficking of lymphocytes into the intestinal mucosa, as a novel class of drugs in the treatment of IBD. In biological therapies against adhesion molecules, Natalizumab and Vedolizumab have been used in the treatment of ulcerative colitis (UC) and Crohn’s disease (CD), which specifically antagonize the α4 integrin to block intestinal mucosal address for cell infiltration^47,48^. In new phase 3 data, subcutaneous vedolizumab with a favorable safety and tolerability profile is effective as maintenance therapy in patients with moderately to severely active ulcerative colitis^47^. ANXA5 administration significantly reduced the inflammatory cell infiltration in TNBS-induced colitis, which was probably due to its inhibitory effect on leukocyte adhesion to endothelial cells (Fig. 6). PS-targeted delivery of ANXA5 to the colon might be a valuable therapeutic strategy in the management of refractory IBD, or inflammation-associated vascular disease.

ANXA5 has already been used safely in patients as a diagnostic tool for atherosclerosis^49^. Although ANXA5 administration showed the accumulation in the liver and kidney of colitic mice, there was no sign for hepatic hypertrophy (Fig. 3). To further assess its side-effect, high dose of ANXA5 at 700 mg/kg was injected i.v. into healthy mice. Body weight is a major marker for the evaluation of treatment-related toxicity in preclinical studies. There were no significant differences in the body weight between two groups of experimental mice (Supplementary Fig. S7a). Notably, H&E staining also showed no significant changes in the sections of liver, kidney, and colon from mice treated with ANXA5 protein at high dosage (Supplementary Fig. S7b), indicating the low toxicity of ANXA5 as protein drug. In conclusion, the externalized PS on the surface of colonic capillaries is a novel target for IBD therapy and ANXA5 treatment is a promising therapeutic approach for patients with IBD that needs to be further studied.

## Materials and methods

### Establishment of experimental colitis

TNBS-induced acute colitis in 6–8-week-old female BALB/c mice was established as reported previously^50^. Briefly, 2 mg of TNBS (Sigma, St Louis, MO, USA) in 100 μl 50% ethanol was slowly administered into the lumen of the colon using a cannula inserted 4 cm through the anus. Mice were randomly divided into six groups: sham control received 50% ethanol alone, vehicle control with injection of 100 μl PBS, ANXA5 groups pretreated with ANXA5 (0.05 or 0.10 mg kg^−1^) at 30 min before TNBS induction and administered intravenously once a day after TNBS induction, and A5m groups (0.05 and 0.10 mg kg^−1^) in the same treatment. Body weight, stool consistency, and the presence of gross blood in feces were monitored and monitored daily. The colons were excised for macroscopic observation, histopathological analysis, and chemokines/cytokines analysis. The disease activity index (DAI) was used to evaluate the grade of intestinal inflammation in TNBS-induced colitis^51^. For histopathological examination, histological score of H&E staining sections was graded from 0 to 4 according to a previous report^52^.

### In vivo imaging

Purified ANXA5-TagRFP (2 mg kg^−1^) was injected into nude mice through the tail vein. After 30 min, in vivo imaging was visualized by an IVIS Lumina XR system (Caliper Life Sciences, Hopkinton, USA) with TagRFP excitation (570 nm) and emission (672 nm) filter sets. After dissecting mice, Ex vivo fluorescence of various tissues was photographed and analyzed for ANXA5-TagRFP. Similarly, BALB/c mice with TNBS-induced colitis were injected by the tail vein with ANXA5-TagRFP (2 mg kg^−1^). After 30 min, mice were dissected to examine the fluorescence intensity of ANXA5-TagRFP in various tissues by an IVIS Lumina XR system and analyzed by Living Image software.

### Inflammatory cell infiltration

EGFP-positive inflammatory cells were recovered from peritoneal fluid of EGFP mice in murine thioglycollate elicited peritonitis model^53^. Mice with TNBS colitis were pretreated with ANXA5 or A5m (0.1 mg kg^−1^) by tail vein injection and then transferred with EGFP^+^ inflammatory cells (10^5^ cells per mouse). After 24 h of injection, the leukocytes in colonic mucosal were collected according to a routine procedure^54^, and then analyzed for EGFP-positive cells by flow cytometer (FACS Calibur, San Jose, CA, USA) equipped with CellQuest software. The colonic sections were counterstained with DAPI for fluorescence microscope observation.

For immunohistochemistry analysis of cell infiltration, colonic sections were blocked with 2.4G2 hybridoma supernatant (1:500; BD Biosciences) and then stained with PE-conjugated rat anti-mouse CD11b (1:500, BD Biosciences). The staining of sections were digitally photographed and quantified by Photoshop software. Cell isolation from the corresponding samples were stained with PE-conjugated rat anti-mouse CD11b antibody and then analyzed by FACS using CellQuest software.

### Cell adhesion assay

The cell adhesion assay was performed as described previously^25^. HUVECs were seeded to confluence in 6-well plates and then stimulated by LPS (0.2 μg/ml, Sigma) for 6 h. Then activated HUVECs were untreated or treated with ANXA5/A5m (250 nM) for 30 min in complete medium supplemented with 2.5 mM Ca^2+^, and labeled with Hoechst (Invitrogen, Carlsbad, CA). THP-1 cells were labeled with CellTracker Green CMFDA (Invitrogen, Carlsbad, CA) and then co-incubated with activated HUVECs for 1 h. Non-adherent cells were washed off by PBS. The cell adhesion of THP-1 was observed at 496 nm excitation and 516 nm emission wavelengths by Zeiss fluorescence microscope. Random microscopic fields (×40) (*n* = 6) were photographed.

### Detection for ANXA5 and TLR4 internalization

For ANXA5 internalization, HUVECs were stimulated with LPS (1 μg/ml) and incubated with ANXA5-EGFP or A5m-EGFP (50 μg/ml) for 30 min in the presence of Ca^2+^ (2.5 mM). The internalization of EGFP-fused protein was visualized by Zeiss fluorescence microscope. For TLR4 internalization, FITC-labeled anti-TLR4 antibody (1:500, Abcam, ab8378) was incubated with LPS-activated HUVECs. Thirty minutes after ANXA5-TagRFP treatment, cells were washed with PBS, then fixed, and stained with DAPI. The images were photographed and analyzed by Zen 2012 software (Zeiss Inc., Germany).

### Statistical analysis

Results are presented as mean ± S.D. Statistical analyses were performed using Graphpad Prism (v6; GraphPad Software, La Jolla, CA, USA). The difference between groups was evaluated by one-way ANOVA and Student *t*-test. A value of *p* < 0.05 was considered significant.

## Supplementary information

Supplemental material

## Data Availability

The data sets used for the current study are available from the corresponding author upon reasonable request.
