# Supplementary material for: Phosphatidylserine externalized on the colonic capillaries as a novel pharmacological target for IBD therapy
Source: Signal Transduct Target Ther. 2021 Jun 16;6:235. doi: 10.1038/s41392-021-00626-z (PMC8206212; doi:10.1038/s41392-021-00626-z)
Supplement: Supplementary file 1 — Supplemental material [file 41392_2021_626_MOESM1_ESM.pdf]

Supplementary Materials for

Phosphatidylserine externalized on the colonic capillaries as a novel pharmacological target for IBD therapy

Xuerui Zhang, Lulu Song, Lin Li, Banghui Zhu, Lina Huo, Zhaoqing Hu, Xinran Wang, Jie Wang, Mengyue Gao, Jing Zhang and Zichun Hua

Correspondence to: jzhang08@nju.edu.cn; zchua@nju.edu.cn

This PDF file includes:

Materials and Methods
Figures. S1 to S7
Tables S1

Materials and methods

Mice and cell lines

Specific pathogen-free, female nude mice (5~6 weeks old, ~20 g), BALB/c mice, and C57BL/6 (6~8 weeks old, 16~18 g) mice were obtained from Model Animal Genetics Research Center of Nanjing University (Nanjing, China) and maintained in pathogen-free conditions. All the animal experiments were approved by Nanjing University Animal Care and Use Committee (NJU-ACUC).

Human umbilical vein endothelial cells (HUVEC), human monocytic-leukemia cells (THP-1) were cultured in RPMI 1640 (Gibco-BRL, Basel, Switzerland) and A549 cells were cultured in Dulbecco's modified Eagle's medium (DMEM) (Gibco-BRL, Basel, Switzerland) containing antibiotics and 10% FCS (Gibco-BRL) supplemented with Penicillin and streptomycin (100 U ml^{-1}, WISENT, Montreal, Canada).

Antibodies

PE-conjugated rat anti-mouse CD11b (#553311) was purchased from BD Biosciences. FITC-labelled anti-TLR4 antibody (#ab8378) was purchased from Abcam. Rabbit anti-PS antibody (#05-719) was purchsed from Merck Millipore. Rabbit anti-MECA 32 (#82489) and rabbit anti-mouse CD11b (#46512) was purchased from Cell Signaling Technology. AF488 and AF594 conjugated secondary antibodies (#A-11008, #A-11012) were purchased from Invitrogen. For western blot, antibodies against P-P65 (#3033)，P65 (#8242)，iKB (#4814)，actin (#4970) were purchased from Cell Signaling Technology.

Staining for PS exposure

Phosphatidylserine exposure was evaluated via intravenous injection of PS-targeting antibody (05-719, Merck Millipore, Darmstadt, Germany). Mice received 100 μg anti-PS antibody or control IgG by tail vein injection. 30 min later, mice were sacrificed and the colons were collected for cryotomy. Then the frozen sections were treated with 3% hydrogen peroxide for 15 min, blocked with 3% goat serum for 1 h, and stained with anti-MECA 32 (1:500) (BD Biosciences, San Jose, CA, USA) at 4 ℃ overnight. After washing and applying secondary antibodies (1:2000 dilutions of AF488 and AF594 conjugated secondary antibodies, Invitrogen, Carlsbad, CA) at room temperature, the samples were counterstained with DAPI (Invitrogen, Carlsbad, CA) and imaged using Zeiss fluorescence microscope (Zeiss AX10, CarlZeiss AG, Germany).

TUNEL assay

The frozen colon tissue sections were fixed with formaldehyde for 15 min at 4 ℃ and washed twice with PBS buffer. Then the sections were incubated with proteinase K solution (Beyotime Biotech, Shanghai, China) at room temperature for 5 minutes, refixed with formaldehyde, and washed twice with PBS again. Next, these the sections were analyzed by a TUNEL BrightRed detecion kit (Vazyme, Nanjing, China) according to the manufacturer's instruction. The positive control was pretreated with Dnase I (Sangon Biotech, Shanghai, China). After incubating with 50 μl DAPI (Invitrogen, Carlsbad, CA) at room temperature for 5 minutes, observe tissue sections by fluorescence microscope.

Purification of recombinant proteins

All the recombinant proteins were expressed and purified according to the protocol described before[1,2]. In brief, *E.coli BL21* with expression vectors were lysed by sonication in Ca^{2+} binding buffer and centrifuged at 12,000 rpm min^{-1} for 20 min. For ANXA5, ANXA5-EGFP, and

ANXA5-TagRFP, the centrifugal pellets were resuspended in EDTA releasing buffer and purified by DEAE-Sepharose CL-6B column. For A5m and A5m-EGFP, the supernatant was purified by DEAE-Sepharose anionic-exchange chromatography and a column (1.5 by 90 cm) of Sephacryl S-200 superfine (Pharmacia, Uppsala, Sweden). The purified recombinant proteins were analyzed by SDS-PAGE and their purity was tested by HPLC (LC-20AT, Shimadzu, Osaka, Japan) with Sepax Zenix SEC-150 (7.8 × 300mm, 3 μm) (Sepax Technologies, Inc., Newark, DE, USA). Endotoxin was removed by Toxin Eraser TM Endotoxin Removal Kit (Genscript, Nanjing, China).

Circular dichroismspectra

Circular dichroism (CD) signals were detected on a Chirascan (Applied Photophysics Ltd., English) at room temperature. In the far ultraviolet (UV) region, the spectra were recorded over a wavelength range of 190 to 250 nm using a 1 mm path-length cuvette at a protein concentration of 7 μM (ANXA5 or A5m). Spectra were acquired at a scan speed of 200 nm min^{-1}, with a 1 nm band width, and a 0.5 s integration time. Each spectrum is an average of three scans.

Microscale thermophoresis (MST)

PS liposomes were prepared by thin film method according to a routine protocol[3]. MST assay was performed as described previously[4]. The fusion protein (ANXA5-EGFP or A5m-EGFP) was resuspended in reaction buffer (PH 7.4, 10 mM Hepes, 140 mM NaCl, 2.5 mM $CaCl_2$). The MST assay was performed in a Monolith NT.115 (Nanotemper Technologies, Munich, Germany) using hydrophilic capillaries (Nanotemper, K003 Monolith™ NT.115 Hydrophobic Capillaries). Thermophoresis was analyzed on a proprietary instrument after titration.

Molecule modeling

Protein modeling and docking analysis were studied on a standalone Discovery Studio platform (Accelrys Inc., San Diego, CA). Crystal structure of ANXA5 (PDB_ID: 2xo2) was downloaded from the Protein Data Bank (PDB, www.pdb.org). The docking studies for binding interaction pattern was analyzed by DS docking software (DS2.4).

Cytokine measurements by qRT-PCR

Total RNA was isolated from THP-1 or HUVEC cells using RNA isolation reagent (Invitrogen, Carlsbad, CA) and treated with Dnase I (Sangon Biotech, Shanghai, China). The expressions of cytokines and proinflammation factor were determined relative to the housekeeper GAPDH by quantitative RT-PCR using HiScript®Q Select RT Super Mix and SYBR Green PCR kit (Vazyme, Nanjing, China) on a StepOne/StepOne Plus TM Real-Time PCR System (Applied Biosystems). Experiments were performed according to the manufacturer's instructions. Primer details are outlined in the Table S1.

Reference:

1 Zhang, X. *et al.* Anti-cancer activity of Annexin V in murine melanoma model by suppressing tumor angiogenesis. *Oncotarget*. **8**, 42602-42612, (2017).

2 Tait, J. F., Smith, C. & Gibson, D. F. Development of annexin V mutants suitable for labeling with Tc(I)-carbonyl complex. *Bioconjugate Chem*. **13**, 1119-1123, (2002).

3 Gauer, J. W. *et al.* Membrane Modulates Affinity for Calcium Ion to Create an Apparent Cooperative Binding Response by Annexin a5. *Biophys J*. **104**, 2437-2447, (2013).

4 van den Bogaart, G., Meyenberg, K., Diederichsen, U. & Jahn, R. Phosphatidylinositol 4,5-Bisphosphate Increases Ca2+ Affinity of Synaptotagmin-1 by 40-fold. *J Biol Chem*. **287**, 16447-16453, (2012).

Figure. S1

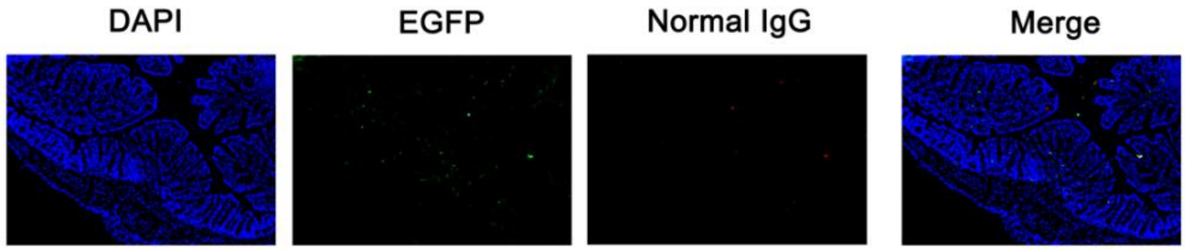

Figure S1. Controls for fluorescence assay in murine colonic sections. Tail vein injection with normal IgG or EGFP as a control for IHC staining. No fluorescence signals were observed.

Figure. S2

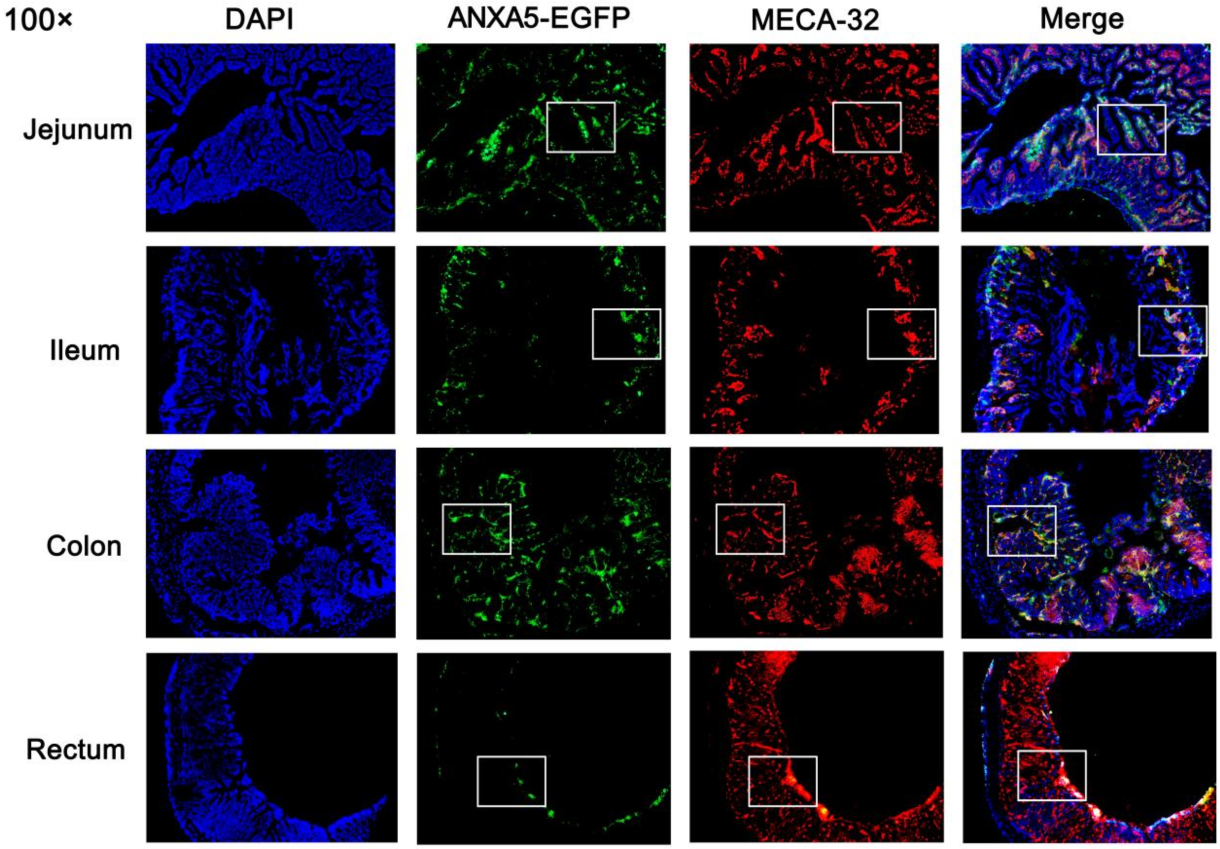

Figure S2. PS exposure on the capillary surfaces of intestinal tissues.
Normal C57BL/6 mice were injected with ANXA5-EGFP by tail vein. The sections of jejunum, ileum, colon and rectum were prepared for immuohistochemical staining with anti-MECA-32 antibody.

Figure. S3

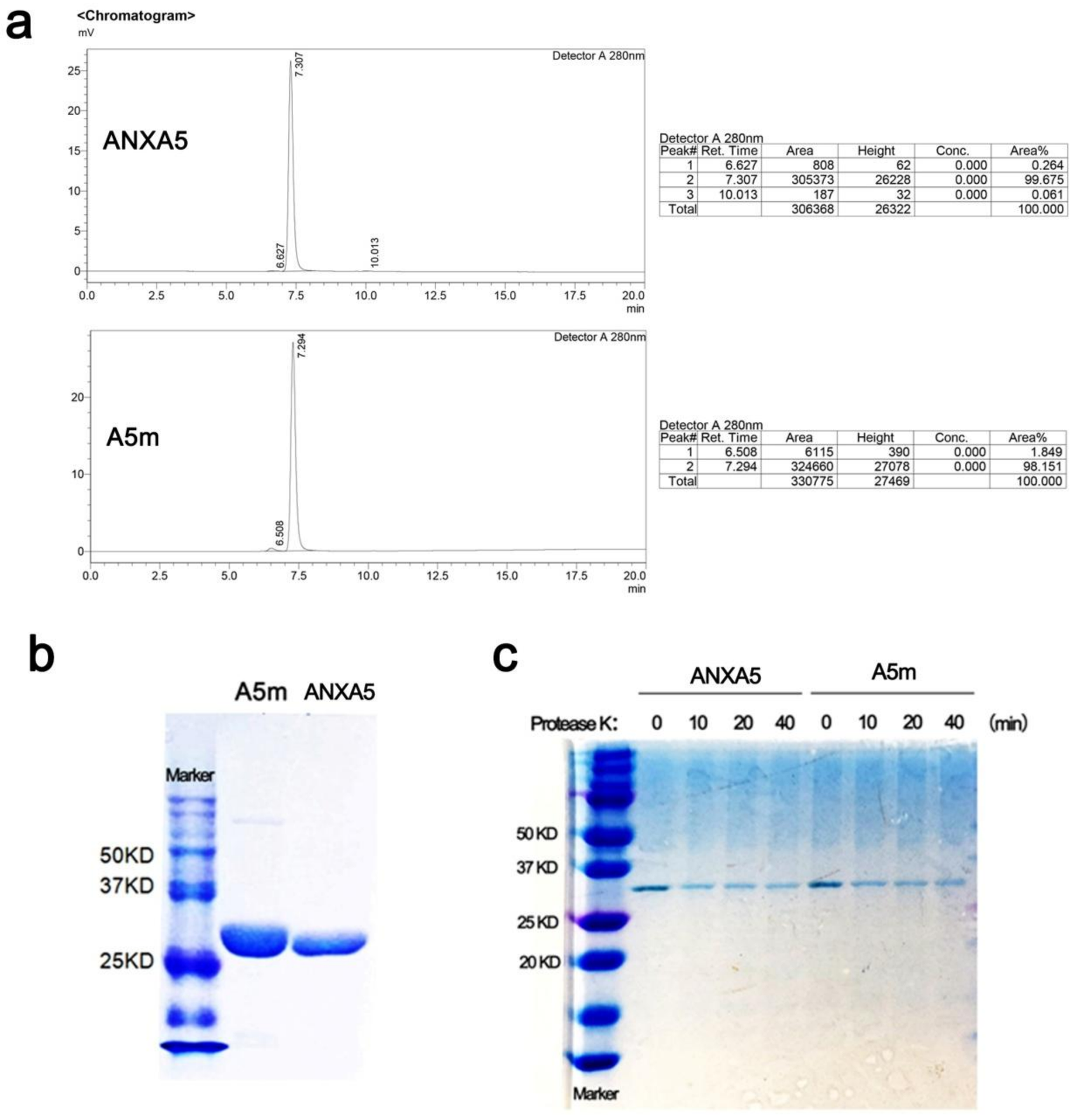

Figure S3. The purity and stability of recombinant proteins ANXA5 and A5m.
The purity of recombinant proteins ANXA5 and A5m were analyzed by HPLC **(a)** and SDS-PAGE **(b)**. After treatment with protease K for different times, the protein stability of ANXA5 and A5m was analyzed by SDS-PAGE **(c)**.

Figure. S4

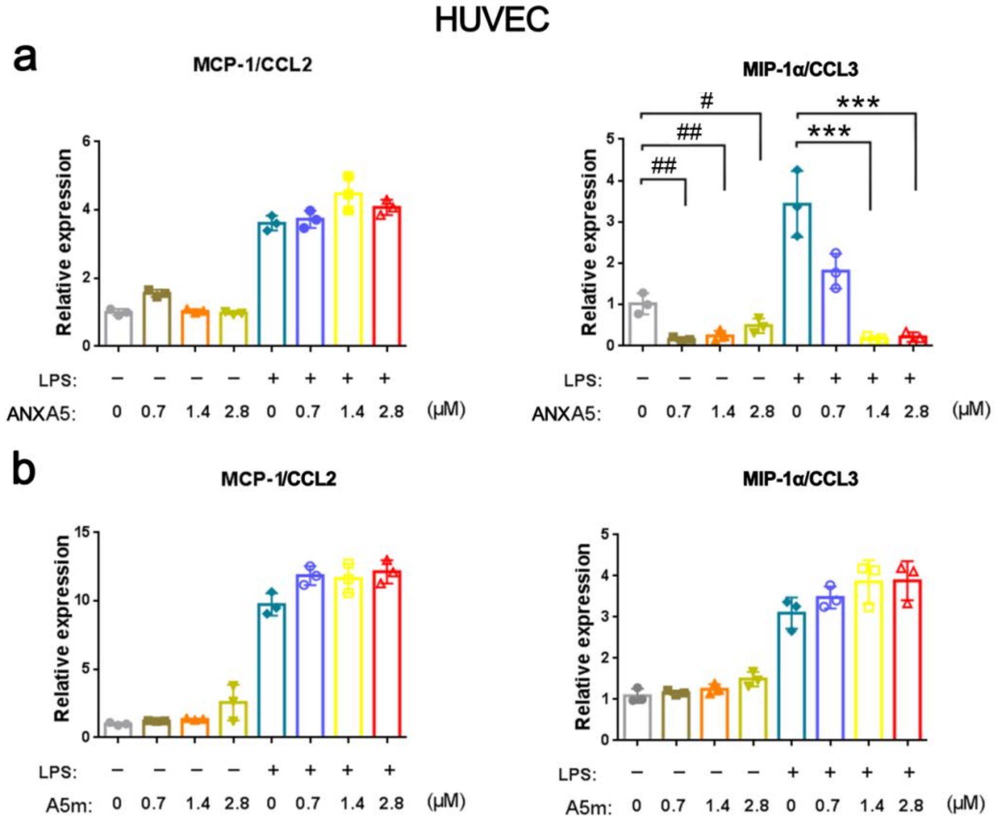

Figure S4. The effect of ANXA5 on the chemokines MCP-1/CCL2 and MIP-1α/CCL3. HUVECs were treated with ANXA5 **(a)** or A5m **(b)** at different doses in the presence or absence of LPS (1 μg/ml). The mRNA levels of MCP-1/CCL2 and MIP-1α/CCL3 were analyzed by qRT-PCR. All data are presented as mean ± SD representative of three independent experiments. *** $P < 0.001$, **** $P < 0.0001$, # $P < 0.05$, ## $P < 0.01$ versus the corresponding control group according to Student's t tests.

Figure. S5

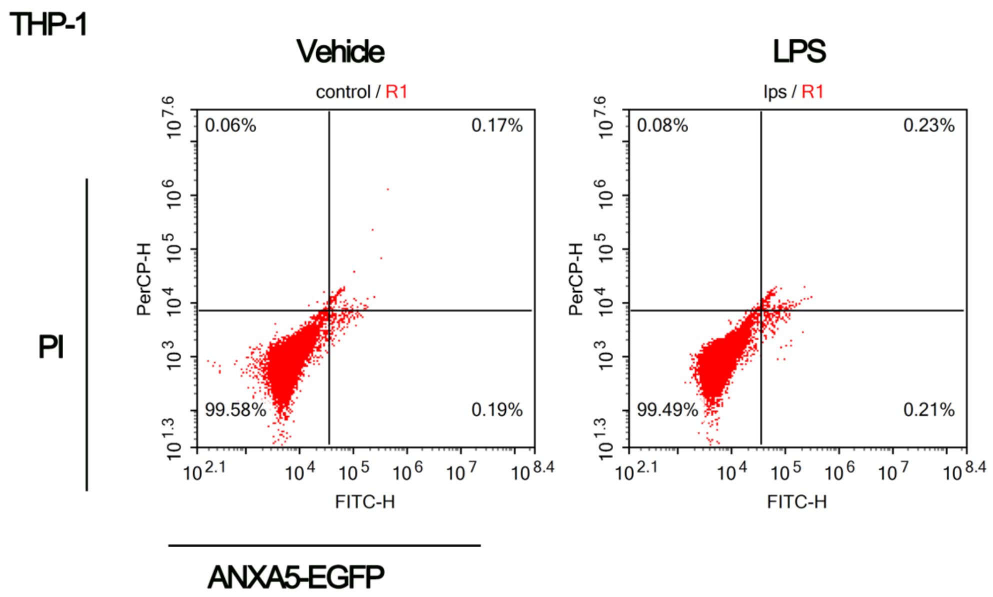

Figure S5. ANXA5 has no binding on THP-1 cells.
THP-1 cells were induced by LPS (10 ng/ml) for 6 h and stained with ANXA5-EGFP for FACS analysis.

Figure. S6

a

Sequences for RT-qPCR

TLR4	Sence	AATGGATCAAGGACCAGAGG
	Antisense	CAGCCAGCAAGAAGCATCAG
ß-actin	Sence	TTGTATCGTGGAAGGACTCA
	Antisense	TGTCATCATATTTGGCAGGTTT

Sequences for siRNA[1]

TLR4	Sence	CGATGATATTATTGACTTA
	Antisense	TAAGTCAATAATATCATCG

b

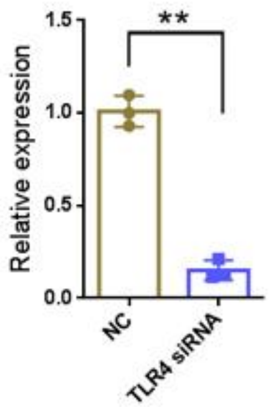

Figure S6. Knockdown of TLR4 in HUVECs.
a) The information of TLR4 sequences for qPCR and RNAi. **b)** HUVECs were transfected with NC siRNAs or TLR4 siRNAs for 36 h. The level of TLR4 mRNA was detected by qRT-PCR. All data are presented as mean ± SD representative of three independent experiments. ** $P < 0.01$ versus the corresponding control group according to Student's t tests.

Reference:

1 Wang, A. C., Su, Q. B., Wu, F. X., Zhang, X. L. & Liu, P. S. Role of TLR4 for paclitaxel chemotherapy in human epithelial ovarian cancer cells. *Eur J Clin Invest* **39**, 157-164, doi:10.1111/j.1365-2362.2008.02070.x (2009).

Figure. S7.

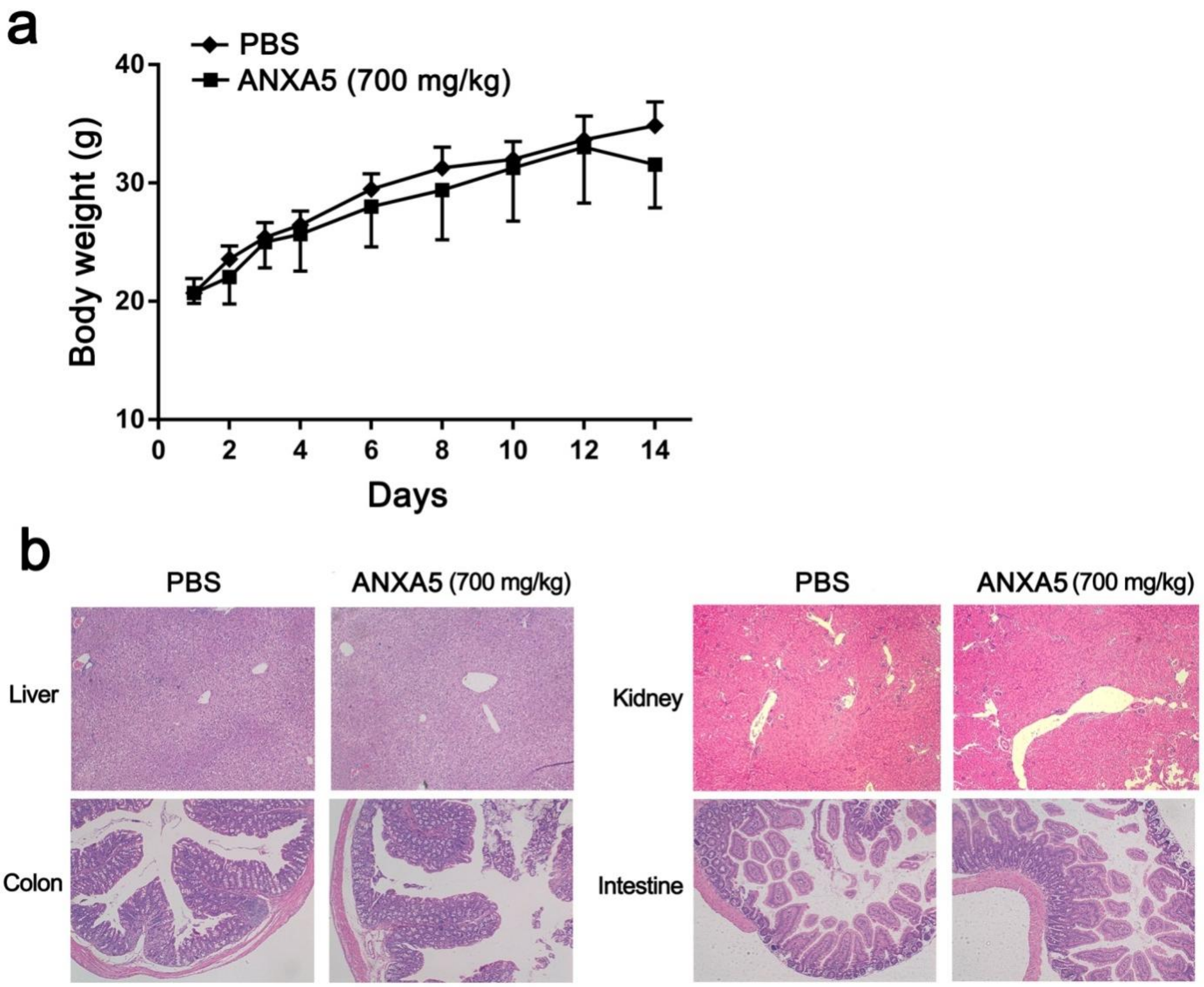

Figure S7. ANXA5 administration with a good biosafety in mice. In the acute toxicity tests, we tried a high dose of ANXA5 at 700 mg/kg in healthy male mice (n=5). **a)** The body weight curve of mice.There were no deaths and no significant differences on weight between the two groups. **b)** Representative images of tissue sections by H&E staining. There were no pathological changes in the liver, kidney, small intestine and colon.

Table S1. Primers

.

Gene	Forward primer (5′→3′)	Reverse primer (5′→3′)
IL-1β	GTGGCAATGAGGATGACTTGTTC	TAGTGGTGGTCGGAGATTCGTA
IL-6	AGCCACTCACCTCTTCAGAAC	GCCTCTTTGCTGCTTTCACAC
IL-8	CTGATTTCTGCAGCTCTGTG	GGGTGGAAAGGTTTGGAGTATG
TNF-α	CTGCTGCACTTTGGAGTGAT	AGATGATCTGACTGCCTGGG
VCAM-1	CGTCTTGGTCAGCCCTTCCT	ACATTCATATACTCCCGCATCCTTC
ICAM-1	AGGCCACCCCAGAGGACAAC	CCCATTATGACTGCGGCTGCTA
MCP-1	CATTGTGGCCAAGGAGATCTG	CTTCGGAGTTTGGGTTTGCTT
MIP-1α	TCGAGCCCACATTCCGTCAC	GCTTTGGTGCCATGACTGCC
GAPDH	TGCACCACCAACTGCTTAGC	GGCATGGACTGTGGTCATGAG

Table S1: Primers used for qRT-PCR analysis.
